# Histomorphometrical Assessment of Sinus Augmentation Using Allograft (Particles or Block) and Simultaneous Implant Placement

**DOI:** 10.1038/s41598-020-65874-5

**Published:** 2020-06-03

**Authors:** Liat Chaushu, Gavriel Chaushu, Roni Kolerman, Marilena Vered, Sarit Naishlos, Joseph Nissan

**Affiliations:** 1 Departments of Periodontology, The Maurice and Gabriela Goldschleger School of Dental Medicine, Tel Aviv University, Tel-Aviv, Israel; 2 Departments of Oral & Maxillofacial Surgery, The Maurice and Gabriela Goldschleger School of Dental Medicine, Tel Aviv University, Tel-Aviv, Israel; 3 Departments of Oral Pathology, The Maurice and Gabriela Goldschleger School of Dental Medicine, Tel Aviv University, Tel Aviv, Israel; 4 Departments of Pedodontology, The Maurice and Gabriela Goldschleger School of Dental Medicine, Tel Aviv University, Tel Aviv, Israel; 5 Departments of Oral Rehabilitation, The Maurice and Gabriela Goldschleger School of Dental Medicine, Tel Aviv University, Tel Aviv, Israel; 6 Departments of Oral & Maxillofacial Surgery, Rabin Medical Center, Beilinson Campus, Petah Tiqva, Israel

**Keywords:** Biological techniques, Medical research

## Abstract

The aim of the present study was to compare the clinical, radiological and histomorphometrical outcome of simultaneous implant placement following augmentation of atrophic maxillary sinuses using allograft (block or particles). Consecutive patients with maxillary residual alveolar ridge height ≤3 mm, scheduled for sinus floor augmentation with simultaneous implant placement, were randomly included. Allograft bone-block or bone-particles served as grafting material. Simultaneously, dental implants were inserted. Biopsies were taken at second stage surgery (after 9 months) for histomorphometric evaluation. Initially 38 sinus augmentations (29 individuals) were allocated for the study. In 4 out of 21(19%) sinuses using particles it was impossible to stabilize the implants and a second stage insertion was preferred, leaving 34 sinuses for histomorphometric evaluation. The difference in the ability to perform simultaneous implant placement was statistically significant (p < 0.05). Ninety implants were inserted simultaneously. All implants osseointegrated. None of the implants was lost up to the end of follow-up time (Range 50–120 months, Mean 74.5 ± 13.5 months). Bone gain radiographically 12.3 ± 1 mm vs. 11.2 ± 1 mm (block vs. particles respectively) and new bone formation histomorphometrically 27.7 ± 15% vs. 32.1 ± 19% (block vs. particles respectively) showed no statistically significant differences between the two groups. Sinus augmentation using allograft (particles or block) and simultaneous implant placement is predictable. All outcome parameters are similar when sinus bone-blocks augmentation is compared to bone-particles augmentation (radiological new bone gain, implant survival, hisomorphometricly new bone formation) despite the ability to stabilize implants, when placed simultaneously with sinus augmentation. Blocks may be advisable when simultaneous implant placement is imperative in cases with residual alveolar bone height ≤3 mm.

## Introduction

Maxillary sinus augmentation with simultaneous implant placement was initially limited to cases with minimum of 4–5 mm alveolar bone height. This arbitrary limit was chosen as the minimum required for initial implant stability^1–3^. Predictability of simultaneous implant placement even in cases with 1 to 2 mm of residual alveolar bone height was reported^1–4^. At the moment, residual alveolar bone height is not the indicator for a simultaneous procedure but rather the ability to achieve primary implant stability^1,4,5^.

Block bone grafts may provide structural rigidity. Such rigidity may contribute to implant stability independent of the residual alveolar bone height^6^. However, the effect of block bone grafts on new bone formation when placed into the sinus in a simultaneous procedure is still to be elucidated.

Comparative (block or particles) animal studies^7,8^ showed that bone-implant-contact (BIC) was significantly greater when block-bone grafts were used. Autografts used to be the gold standard for grafted bone blocks^6^. A major drawback for using autografts is donor site morbidity^9–11^. Elimination of donor site morbidity encouraged clinicians to use allograft, yielding high survival rates^12–19^.

The present study assessed the ability of simultaneously implant placement and histomorphometric outcome following the augmentation of extremely (residual alveolar ridge height ≤3 mm) atrophic maxillary sinuses with using allograft (block or particles).

## Methods

Consecutive patients, in good health, who presented at the Tel Aviv University Dental School Clinic, and authors’ private practices with maxillary posterior sinus floor bone alveolar height deficiency (≤ 3 mm) measured from pre-operative computerized tomography (CT) were included. All procedures were fully explained to the patients who signed an informed consent, and the Ethics Committee of the Tel Aviv University approved the study protocol. The authors have read the Helsinki Declaration and have followed the guidelines in this investigation.

Under local anesthesia (lidocaine 2%, epinephrine 1:100000, Teva Pharmaceuticals, Petah-Tiqva, Israel), lateral wall access to the maxillary sinus was initiated. The sinus membrane was separated from the sinus floor and implant osteotomies were completed. A cancellous bone-block allograft (ReadiGraft®, Canblock 1.5, Lifenet, Virginia Beach, VA, USA) or cancellous bone-allograft particles (Oragraft®, Lifenet, Virginia Beach, VA, USA) were used. In cases using blocks, the prefabricated grafted block (1.5 ×1.5 ×3.0 cm) was soaked in sterile saline for at least 45 minutes before placement and trimmed with a high-speed bur until adjusted to the lateral opening size. It was then inserted in a gentle press-fit fashion to the sinus cavity prepared area. The cancellous block allograft was pushed maximally up to the palatal wall of the sinus cavity. In most cases it reached two-thirds of its prefabricated depth. Stability of the block was achieved with the window frame. Implant sites were marked using a surgical stent and the osteotomies were performed according to the manufacturers’ recommendation. In cases using cancellous bone-allograft particles, grafting material was placed in the created cavity adapting the grafting material to the bony walls. Implants were inserted in the final position. Collagen membrane (Ossix®Plus, Datum Dental, Lod, Israel) served to cover the lateral bony wall in all the cases. The mucoperiostal flap was closed. Second stage surgery was performed 9 months after sinus augmentation with simultaneous implant placement. The reflection of the buccal flap was extended to the peripheral augmentation area. The previous window location was identified using bone measurements performed during insertion of the grafting material. By careful orientation a trephine bur was used to collect cylindrical sample cores 6–8 mm in depth from the grafting material (block or particles). The trephine was always aimed to the area between the implants to avoid any contact. All specimens were prepared for histological examination.

Healing abutments were placed. Four-six weeks later, impressions were made and the implants were restored with fixed partial metallic restorations.

Oral hygiene maintenance was performed every 3 months. Follow-up appointments were scheduled every six months. Once a year a panoramic radiograph was done and bone gain was evaluated.

### Histomorphometric evaluation

After each sample collection the cores obtained were placed in 10% buffered formalin for a consecutive day. The first laboratory action was placement for 3 days in EDTA, pH = 6 for rapid decalcification. After 4 days the samples were placed in paraffin for cutting. Standard hematoxylin and eosin stained staining was followed. Digital photographs of each bony core histological slide were taken by a digital camera (Olympus BH-2, Tokyo, Japan) placed over a light microscope (Olympus BH-2, Tokyo, Japan). The photomicrographs were saved as jpeg files. A power point presentation was prepared in which each case was represented by 5 successive slides. The entire area of the cores was covered by 5 non overlapping photographs at ×200 magnification. A 10 × 10 grid was superimposed over each photograph for counting. Each time one of these parameter (new bone, graft, connective tissue) was marked. Every field was summarized for each parameter. The mean results of all fields were used as volume fraction (%).

### Statistical analysis

Descriptive presentation of the data included mean and standard deviation. The Student t test and Fisher’s exact test were used for statistical analysis of the data calculated. Independent variable was physical form of bone graft (block vs. particles). The dependent variables were simultaneous implant placement, bone gain, new bone formation, implant survival.

### Ethical approval

All procedures performed in this study are in accordance with the ethical standards of the institutional and/or national research committee and with the 1964 Helsinki declaration and its later amendments or comparable ethical standards.

### Ethics

All participants signed a consent form after being fully informed about the nature of the procedure. The ethics committee of Tel-Aviv University approved the study protocol.

### Informed consent

Informed consent was obtained from all individual participants included in the study.

## Results

Twenty nine patients (12 males and 17 females) and 38 sinuses were included initially in the study (Table 1). In 8 patients, bilateral sinus augmentation was performed. Age ranged 39–74 years, mean 55.5 ± 10 years.

**Table 1:** Patient characteristics.

No. of patients	29
No. of sinuses	38
**Age**	
Range (years)	39–74
Mean (years)	55.5 ± 10
**Gender**	
Male	12
Female	17
No. of implants	90
**Implant diameter (mm)**	
Mean	4.4 ± 0.5
Range	3.75–5
**Implant length (mm)**	
Range	11.5–13
Mean	12.41 ± 1
**Follow-up after implant restoration delivery (months)**	
Range	50–120
Mean	74.5 ± 13.5

In all the sinuses using a bone-block as grafting material, a simultaneous approach was feasible and implant stability was achieved. In 4 out of 21 sinuses using bone-particles as grafting material a simultaneous implant placement was not feasible. It was impossible to stabilize the implants and a second stage implant insertion was performed 6 months later. Sinus augmentation with simultaneous implant placement yielded 81% vs. 100% success in the bone-particles vs. bone block group respectively. The difference was statistically significant (p < 0.05).

Outcome parameters were analyzed for seventeen sinus augmentations in each group.

Radiographic bone gain was similar − 12.3 ± 1 mm vs. 11.2 ± 1 mm (block vs. particles, respectively). The bone volume gained enabled the placement of implants with a mean length of 12.41 ± 1 (range 11.5–13 mm) and a mean diameter of 4.4 ± 0.5 (range 3.75–5 mm) (Table 1).

Implant survival - A total of 90 implants were inserted simultaneously. All implants osseointegrated. None of the implants failed up to the end of follow-up time (Range 50–120 months, Mean 74.5 ± 13.5 months) (Table 1).

**Figure 1:**
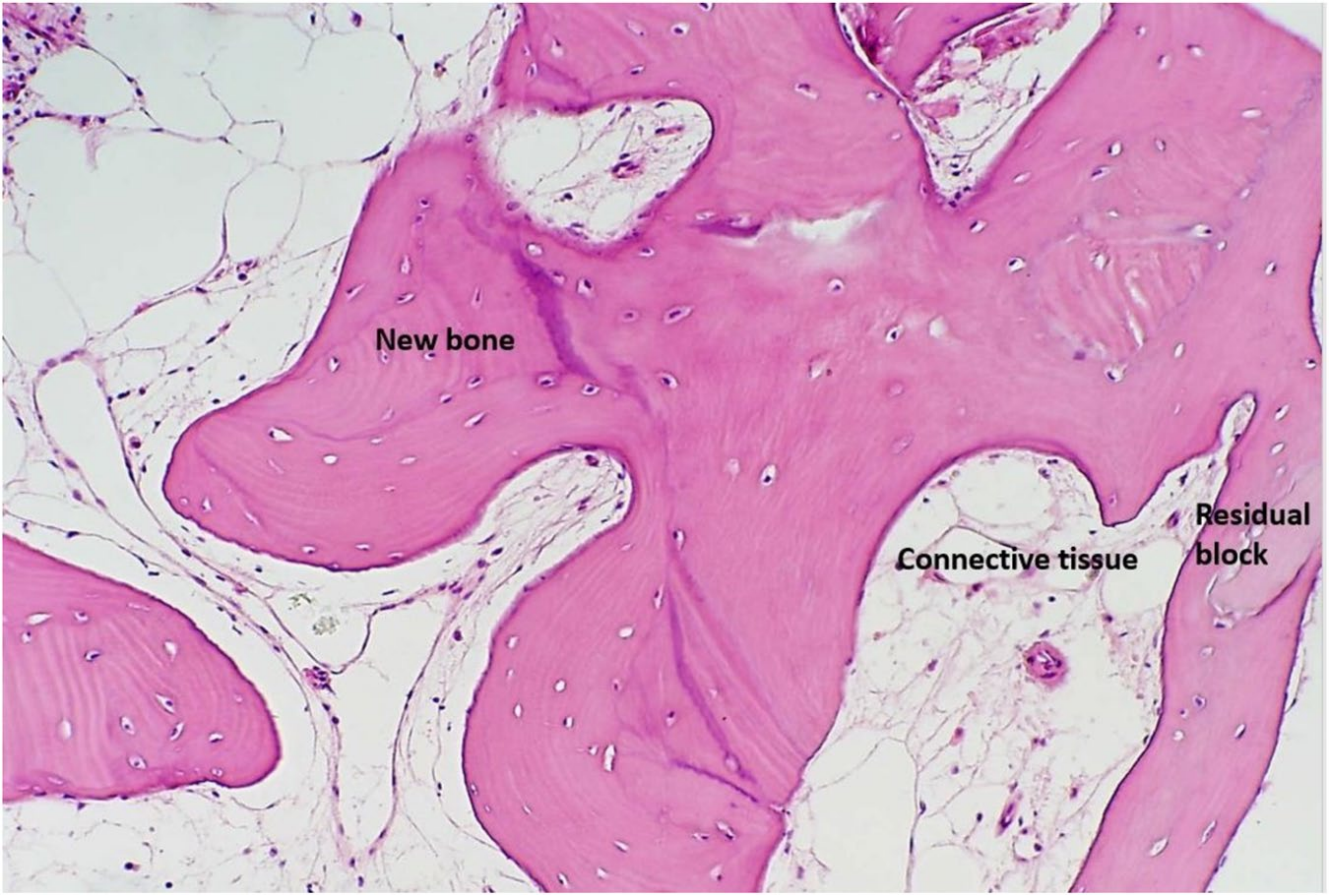
Photomicrograph of a bone biopsy when block-graft was used. Residual block allograft in close contact with newly formed bone (Osteocyte in lacuna) and connective tissue. (Hematoxylin -Eosin staining ×200 magnification).

**Figure 2:**
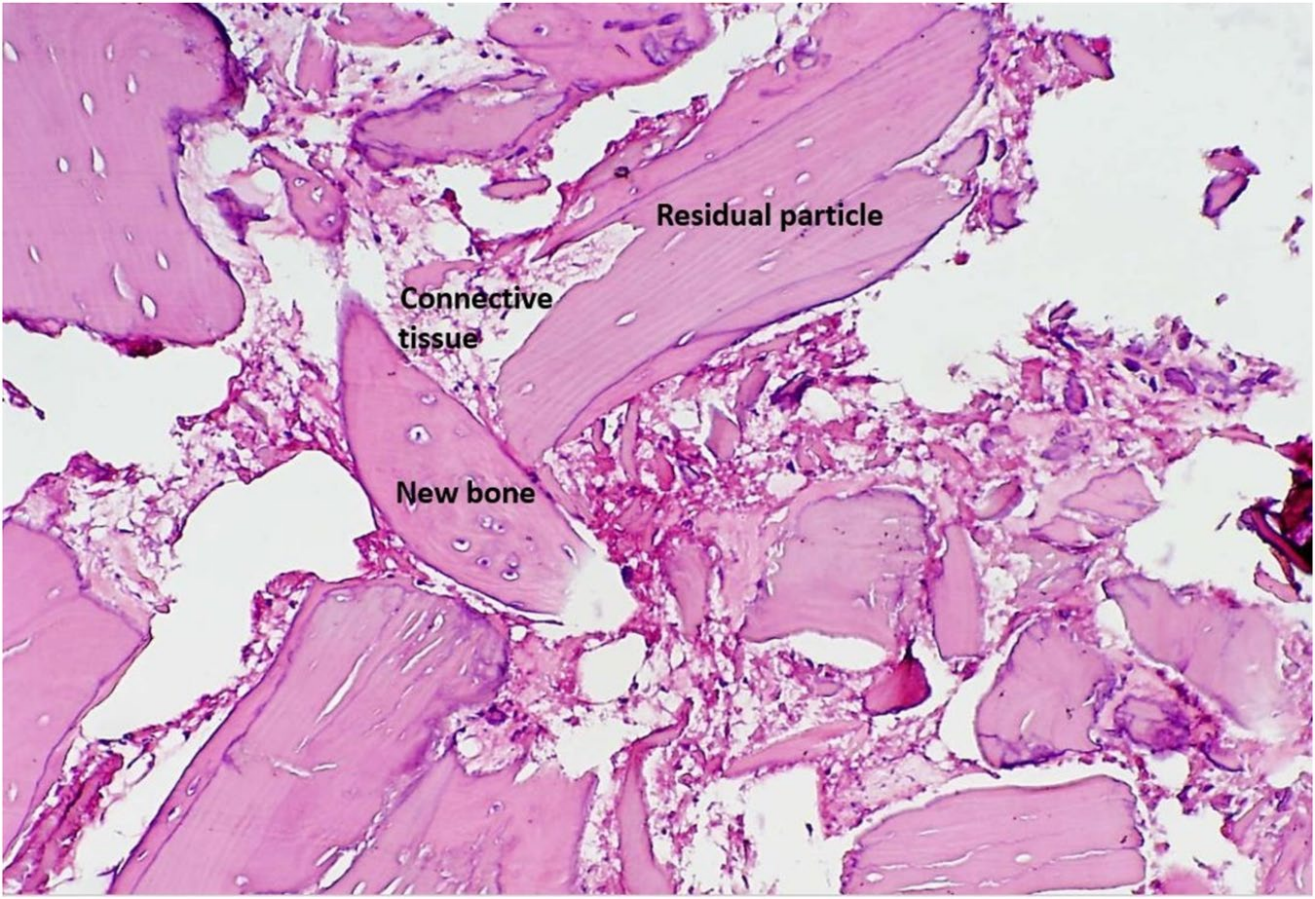
Photomicrograph of a bone biopsy when particulated-graft was used. Residual allograft particles in close contact with newly formed bone (Osteocyte in lacuna) and connective tissue. (Hematoxylin -Eosin staining ×200 magnification).

Histolomorphometricly new bone formation was similar − 27.7 ± 15% vs. 32.1 ± 19%. (block vs. particles respectively) (Figs. 1, 2).

## Discussion

The study assessed the amount of new bone formation around dental implants placed simultaneously using bone allograft (block or particles) for maxillary sinus augmentation in extremely atrophic cases (residual alveolar ridge height ≤3 mm). New bone formation did not differ between the two physical forms of grafting materials.

The present histomorphometric results are in accordance with the reported range for new bone formation^20^. Lee *et al*. 2007^7^, using autogenous bone graft, reported mean BIC of 56.7% vs. 32.1% and mean radiological bone gain of 12.3 mm vs. 9.7 mm (block vs. particles p < 0.05). They concluded that blocks may be superior for sinus floor augmentation.

Simultaneous implant placement (avoiding an additional surgery, thus reducing patient morbidity and chair time) may be advantageous in certain conditions (e.g. minimizing general anesthesia appointments, medically compromised patients, dental phobia, elderly patients, incidence of large Schneiderian membrane tears). The major inclusion criteria of the present study were residual alveolar ridge height ≤3 mm. The use of bone-particles as grafting material in the present study yielded only 81% success for simultaneous implant placement vs. 100% when blocks were used.

The number of missing posterior teeth that needs to be replaced and maxillary atrophy (residual alveolar ridge height) commonly determine the dimensions of the lateral window and the amount of augmentation required. It may be hypothesized that bone healing could be jeopardized in situations where a large window is prepared. It might be assumed that the use of block-allografts for sinus augmentation dictates the creation of a large lateral window. In the present study, inclusion criterion is are already atrophic sinus. Hence, lateral window size is relatively bigger compared to less atrophic cases. Moreover, the use of allogeneic block allows the operator to chose the proper block size, trim and adapt to the minimum required for the specific lateral widow performed. Furthermore, all outcome parameters (radiological new bone gain, hisomorphometricly new bone formation, implant survival, long term follow-up) in the present study were similar for both allogeneic physical forms of bone grafting material.

The major drawback for using block-grafts is the need to trim and adapt the block to the sinus and window dimensions. This may be time-consuming and challenging.

**Figure 3:**
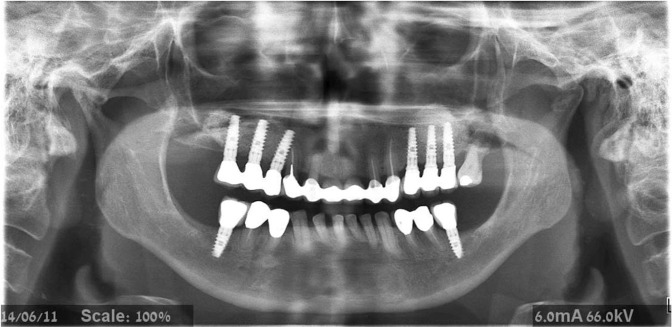
Panoramic radiograph demonstrating block-graft on the right side and particulate graft on the left side of the maxilla.

Despite the fact that particulate bone substitutes may achieve good results when used in sinus augmentation in general, bone-block allografts may offer more structural benefits in extremely atrophic cases (residual alveolar ridge height ≤3 mm), and can be shaped to match the host site^6^. In addition, block grafts may improve primary stability, advantageous for proper osseointegration and simultaneous implant placement^21^ (Fig. 3).

## Conclusions

Sinus augmentation using allograft (particles or block) and simultaneous implant placement is predictable. All outcome parameters are similar when sinus bone-blocks augmentation is compared to bone-particles augmentation (radiological new bone gain, hisomorphometricly new bone formation, implant survival, long term follow-up) despite the ability to stabilize implants, when placed simultaneously with sinus augmentation. Blocks may be advisable when simultaneous implant placement is imperative in cases with residual alveolar bone height ≤3 mm.
