# Author Correction: Histomorphometrical Assessment of Sinus Augmentation Using Allograft (Particles or Block) and Simultaneous Implant Placement

**DOI:** 10.1038/s41598-021-00198-6

**Published:** 2021-10-13

**Authors:** Liat Chaushu, Gavriel Chaushu, Roni Kolerman, Marilena Vered, Sarit Naishlos, Joseph Nissan

**Affiliations:** 1 Departments of Periodontology, The Maurice and Gabriela Goldschleger School of Dental Medicine, Tel Aviv University, Tel-Aviv, Israel; 2 Departments of Oral & Maxillofacial Surgery, The Maurice and Gabriela Goldschleger School of Dental Medicine, Tel Aviv University, Tel-Aviv, Israel; 3 Departments of Oral Pathology, The Maurice and Gabriela Goldschleger School of Dental Medicine, Tel Aviv University, Tel Aviv, Israel; 4 Departments of Pedodontology, The Maurice and Gabriela Goldschleger School of Dental Medicine, Tel Aviv University, Tel Aviv, Israel; 5 Departments of Oral Rehabilitation, The Maurice and Gabriela Goldschleger School of Dental Medicine, Tel Aviv University, Tel Aviv, Israel; 6 Departments of Oral & Maxillofacial Surgery, Rabin Medical Center, Beilinson Campus, Petah Tiqva, Israel

Correction to: *Scientific Reports* 10.1038/s41598-020-65874-5, published online 03 June 2020

The original version of this Article contained an error in the spelling of the author Sarit Naishlos, which was incorrectly given as Sarit Naishols.

The original Article has been corrected.

